# A data-driven approach links microglia to pathology and prognosis in amyotrophic lateral sclerosis

**DOI:** 10.1186/s40478-017-0424-x

**Published:** 2017-03-16

**Authors:** Johnathan Cooper-Knock, Claire Green, Gabriel Altschuler, Wenbin Wei, Joanna J. Bury, Paul R. Heath, Matthew Wyles, Catherine Gelsthorpe, J. Robin Highley, Alejandro Lorente-Pons, Tim Beck, Kathryn Doyle, Karel Otero, Bryan Traynor, Janine Kirby, Pamela J. Shaw, Winston Hide

**Affiliations:** 1 Sheffield Institute for Translational Neuroscience, University of Sheffield, 385A Glossop Road, Sheffield, UK; 2 Department of Biostatistics, Harvard School of Public Health, Boston, USA; 3 Department of Genetics, University of Leicester, Leicester, UK; 4 Biogen Inc, 115 Broadway, Cambridge, USA; 5 Neuromuscular Diseases Research Section, Laboratory of Neurogenetics, NIA, NIH, Bethesda, USA

**Keywords:** Transciptome, Microglia, Amyotrophic lateral sclerosis, Neuropathology, TREM2, Biomarkers

## Abstract

Amyotrophic lateral sclerosis (ALS) is a devastating neurodegenerative disease that lacks a predictive and broadly applicable biomarker. Continued focus on mutation-specific upstream mechanisms has yet to predict disease progression in the clinic. Utilising cellular pathology common to the majority of ALS patients, we implemented an objective transcriptome-driven approach to develop noninvasive prognostic biomarkers for disease progression. Genes expressed in laser captured motor neurons in direct correlation (Spearman rank correlation, *p* < 0.01) with counts of neuropathology were developed into co-expression network modules. Screening modules using three gene sets representing rate of disease progression and upstream genetic association with ALS led to the prioritisation of a single module enriched for immune response to motor neuron degeneration. Genes in the network module are important for microglial activation and predict disease progression in genetically heterogeneous ALS cohorts: Expression of three genes in peripheral lymphocytes - *LILRA2*, *ITGB2* and *CEBPD* – differentiate patients with rapid and slowly progressive disease, suggesting promise as a blood-derived biomarker. TREM2 is a member of the network module and the level of soluble TREM2 protein in cerebrospinal fluid is shown to predict survival when measured in late stage disease (Spearman rank correlation, *p* = 0.01). Our data-driven systems approach has, for the first time, directly linked microglia to the development of motor neuron pathology. *LILRA2*, *ITGB2* and *CEBPD* represent peripherally accessible candidate biomarkers and TREM2 provides a broadly applicable therapeutic target for ALS.

**Electronic supplementary material:**

The online version of this article (doi:10.1186/s40478-017-0424-x) contains supplementary material, which is available to authorized users.

## Introduction

Amyotrophic lateral sclerosis (ALS) is a neurodegenerative disease without effective treatment or a predictive biomarker [50]. Progressive motor neuron loss leads to a median survival of only 32 months, with death most often the result of respiratory failure [42]. A useful non-invasive biomarker for ALS progression should anticipate disease severity in advance, be broadly applicable independent of genetic background, and should provide a basis for therapeutic intervention. Many biomarkers in development currently are potentially limited because they are phenomenological including electrophysiological [14], imaging [19], clinical [41] and fluid-based [29] measures. Other upstream markers are specific to relatively rare genetic variants e.g. RNA foci and dipeptide-repeat proteins in *C9ORF72*-ALS [8, 30].

**Fig. 1:**
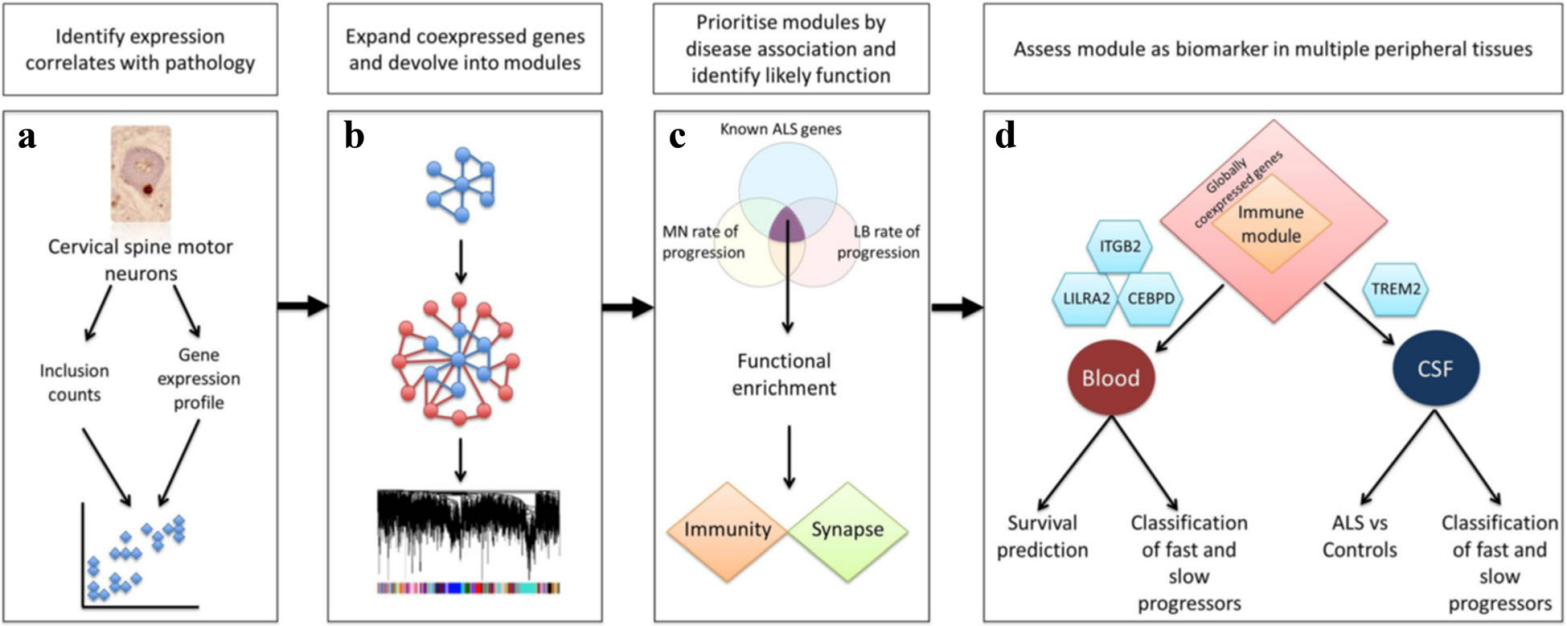
Data-driven discovery workflow. Using anterior horn tissue, RNA transcript expression was measured from isolated motor neurons, and counts of p62-positive cytoplasmic inclusions within motor neurons were conducted. RNA expression and pathology counts from the same patients were correlated by Spearman’s rank correlation to identify 83 transcripts (**a**). Pathology correlated transcripts seeded co-expressed networks. The resulting combined network was developed into tightly co-expressing modules using weighted gene co-expression analysis (WGCNA) (**b**). Modules were prioritised using enrichment with independently curated gene lists related to ALS rate of progression and ALS genetic susceptibility. The two top scoring modules were enriched for neuronal and immune function respectively. MN = motor neuron, LB = lymphoblastoid (**c**). The immune module was selected for use as a biomarker in peripheral tissue and additional non-tissue specific genes were added. Components of the immune module were assessed by mRNA and protein quantification for predictive value in blood and cerebrospinal fluid (CSF) (**d**)

Data-driven methods employing transcriptomics have successfully identified biomarkers in other clinically and genetically heterogeneous disease states, including breast and gastric cancers, psoriasis and progressive supranuclear palsy [26, 43, 48, 54]. To achieve a similar goal we planned an approach with a discovery phase followed by a biomarker phase. In the discovery phase, we utilised a systematic, data-driven approach to discover and prioritise modules of tightly co-expressed genes relevant to ALS pathogenesis (Fig. 1a–c). In the biomarker assessment phase we tested the capability of top performing modules as a biomarker (s) when measured in accessible tissue (Fig. 1d).

In order to deconstruct central nervous system (CNS) pathophysiology, studies have concentrated on a single dysfunctional feature, an approach that may not yield sufficiently broad insights into global disease mechanisms. An advantage of a global transcriptome-based analysis is the capacity to exhaustively describe a biological system without prior information [28]. Integrating transcriptome profiling with measurement of a disease-specific covariate reveals the contribution of individual genes to pathogenesis [22]. Extending this concept to the level of networks of interacting genes rather than isolated genes provides further physiological insight [17].

The aim of our approach is to develop non-invasive, broadly applicable prognostic biomarkers for ALS disease progression. Although ALS is markedly heterogeneous both genetically and phenotypically, more than 98% of ALS patients develop p62- and TDP-43-positive neuronal cytoplasmic inclusions within degenerating motor neurons [33]. Post-mortem studies have indicated that the frequency of neuronal TDP-43-positive cytoplasmic aggregates predicts the severity of neurodegeneration in a region-specific manner [2]. We selected motor neuron pathology as a covariate measure of disease severity which could potentially be used to identify important, broadly applicable, transcriptome changes related to outcome.

To maximize signal from the relevant affected system, gene expression profiling was performed on laser captured motor neurons from ALS patients. Genes correlated in their levels of expression with motor neuron pathology were then developed into co-expression network modules which were filtered and prioritised based on independently curated markers of ALS biology: gene sets related to rate of progression and upstream genetic association with ALS. In the discovery phase (Fig. 1a–c) our systematic approach led to identification of two gene modules enriched with ALS biology. Functional enrichment within the top scoring network module revealed genes which encode an immune response to motor neuron pathology; the majority of these genes are expressed by microglia.

Gene expression within the CNS has been observed in peripheral tissues [18, 22] so in the biomarker assessment phase (Fig. 1d) of our analysis, we explored the possibility that our modules, generated from CNS tissue, may include genes with tissue-independent ability to predict disease severity. Components of the immune module were assessed by mRNA and protein quantification in accessible tissues such as blood and cerebrospinal fluid (CSF). We demonstrate candidate biomarkers that provide insight into potential therapeutic targets.

## Materials and methods

### Laser captured motor neurons

Brain and spinal cord tissue from fourteen ALS patients was obtained from the Sheffield Brain Tissue Bank (Table 1). Seven of these patients carried a hexanucleotide repeat expansion of *C9ORF72* and seven patients suffered sporadic ALS with no identified pathogenic mutation. *C9ORF72*-ALS samples were identified by repeat-primed PCR of the *C9ORF72* gene [9]. Common mutations in *C9ORF72*, *TARDBP*, *FUS*, *CHMP2B* and *SOD1* were excluded in the sporadic ALS patients. Tissue donated for research was obtained with written informed consent from the next of kin, and in accordance with the UK Human Tissue Authority guidelines on tissue donation. The work was approved by the South Yorkshire Ethics Committee.

**Table 1:** Clinical information relating to motor neurons laser captured from ALS patients

ID	Gender	Age at onset (years)	Disease duration (years)	Site of onset	*C9ORF72* status	Familial/Sporadic
1	M	70	2.17	Limb	+	Familial
2	M	66	1.17	Bulbar	+	Familial
3	F	56	3.58	Limb	+	Familial
4	F	61	3.33	Bulbar	+	Sporadic
5	F	58	0.58	Limb	+	Sporadic
6	M	62	1.67	Bulbar	+	Sporadic
7	F	61	3.50	Limb	+	Sporadic
8	M	60	3.17	Limb	-	Sporadic
9	M	71	0.75	Limb	-	Sporadic
10	M	49	2.42	Limb	-	Sporadic
11	M	74	5.25	Limb	-	Sporadic
12	M	71	4.00	Limb	-	Sporadic
13	M	69	3.92	Limb	-	Sporadic
14	M	50	2.08	Limb	-	Sporadic

C9ORF72 status indicates the presence (+) or absence (-) of a GGGGCC-repeat expansion in the C9ORF72 gene

*M* Male, *F* Female

Spinal cord sections from the limb enlargements were collected postmortem, processed according to standard protocols [21], and stored at −80 °C until required. Cervical spinal cord sections were prepared, between 800 and 1200 motor neurons were isolated, and RNA was extracted using methods described previously [15]. RNA quantity and quality was assessed on the Nanodrop spectrophotometer and Agilent Bioanalyser, respectively, to ensure all samples were of comparable and sufficient quality to proceed. RNA (20–25 ng) was linearly amplified using the Affymetrix Two Cycle cDNA synthesis protocol to produce biotin-labelled copy RNA. Copy RNA (15 μg) was fragmented for 15 min and hybridized to the Human Genome U133 Plus 2.0 GeneChips, according to Affymetrix protocols. Array washing and staining was performed in the GeneChip® fluidics station 400 and arrays were scanned on the GeneChip® 3000 scanner. GeneChip® Operating Software was used to generate signal intensities for each transcript.

### Lymphoblastoid cell lines

Lymphoblastoid cell lines derived from 46 Caucasian ALS patients, all of Northern European descent, were obtained from the UK Motor Neurone Disease Association DNA Bank (Table 2). *C9ORF72*-ALS samples were identified by repeat-primed PCR of the *C9ORF72* gene [9]. All samples were collected with written informed consent from the donor, and the work was approved by the South Yorkshire Ethics Committee.

**Table 2:** Clinical information relating to lymphoblastoid cell lines derived from ALS patients

ID	Gender	Age at onset (years)	Disease duration (years)	Site of onset	*C9ORF72* status	Familial/Sporadic
1	F	28	1.10	Bulbar	+	Familial
2	F	57	1.21	Mixed	+	Familial
3	F	62	0.17	Bulbar	+	Familial
4	M	59	<1 year	N/A	+	Familial
5	M	63	1.71	Mixed	+	Familial
6	M	47	1.63	Limb	+	Familial
7	F	51	0.97	Bulbar	+	Familial
8	M	60	1.15	Bulbar	+	Sporadic
9	M	68	1.56	Limb	+	Sporadic
10	F	37	1.74	Limb	+	Sporadic
11	M	56	2.20	Limb	+	Sporadic
12	M	45	1.47	Limb	+	Sporadic
13	M	72	0.52	Limb	+	Sporadic
14	F	58	1.33	Mixed	+	Sporadic
15	M	47	1.57	Limb	+	Sporadic
16	M	64	0.66	Limb	+	Sporadic
17	M	62	1.96	Limb	+	Sporadic
18	M	65	1.40	Limb	+	Sporadic
19	F	69	>4 years	Limb	+	Familial
20	M	63	>5 years	Limb	+	Familial
21	F	64	6.92	Limb	+	Familial
22	F	56	4.14	Limb	+	Familial
23	F	72	4.66	Limb	+	Sporadic
24	F	48	5.95	Limb	+	Sporadic
25	F	37	4.50	Bulbar	+	Sporadic
26	M	61	~4 years	Mixed	+	Sporadic
27	M	44	0.94	Mixed	-	Sporadic
28	M	54	5.90	Limb	-	Sporadic
29	M	72	5.99	Limb	-	Sporadic
30	M	76	0.85	Limb	-	Sporadic
31	F	76	1.08	Bulbar	-	Sporadic
32	F	65	1.10	Limb	-	Sporadic
33	M	65	7.70	Limb	-	Sporadic
34	M	49	0.39	Limb	-	Sporadic
35	M	32	6.05	Limb	-	Sporadic
36	M	35	6.85	Limb	-	Sporadic
37	F	62	0.73	Bulbar	-	Sporadic
38	F	58	6.02	Limb	-	Sporadic
39	M	66	0.85	Limb	-	Sporadic
40	M	82	6.67	Limb	-	Sporadic
41	F	75	0.89	Limb	-	Sporadic
42	M	49	0.70	Limb	-	Sporadic
43	F	68	6.17	Limb	-	Sporadic
44	F	62	6.69	Limb	-	Sporadic
45	M	71	7.12	Limb	-	Sporadic
46	M	86	1.04	Limb	-	Sporadic

C9ORF72 status indicates the presence (+) or absence (-) of a GGGGCC-repeat expansion in the C9ORF72 gene

*M* Male, *F* Female

Total RNA was extracted from ALS patient and control-derived lymphoblastoid cell lines using QIAGEN’s RNeasy® Mini Kit following the manufacturer’s recommendations. A 75 μL LCL suspension, containing approximately 5x10^6^ cells, typically yields between 1.9 and 13.6 μg total RNA with a mean concentration of approximately 170 ng/μl as assessed the by the NanoDrop 1000 spectrophotometer (Thermo Scientific). The quality of the isolated material was analysed using the 2100 bioanalyzer with an RNA 6000 Nano LabChip® Kit (Agilent Technologies, Inc.). Linear amplification of RNA with an input of approximately 300 ng of starting material was performed using the Ambion® Whole Transcript (WT) Expression Assay (*Applied Biosystems*) and Affymetrix GeneChip® WT Terminal Labelling Kit. This procedure generated fragments of biotin-labelled sense-stranded copy DNA (6–10 μg) between 40 and 70 nucleotides in length that were hybridized onto Human Exon 1.0ST GeneChip® Arrays according to Affymetrix protocols. Array washing, staining and visualisation were performed as described for motor neuron derived RNA.

### Immunohistochemistry

Cervical spinal cord anterior horn was examined from 11 ALS patients including seven *C9ORF72*-ALS patients and four patients with sporadic ALS (Table 1, patients 1–11). Immunohistochemistry was performed for p62 and phospho-TDP-43.

In staining for p62, slides were first deparaffinised through two changes of xylene and hydrated through decreasing concentrations of alcohol (2×100%/1x95%/1x70%). Antigen retrieval was achieved by boiling the samples in trisodium citrate at pH 6.5, and endogenous peroxidase was blocked in 3% H_2_O_2_ in methanol for 20 min. The slides were then stained using the VECTASTAIN Elite ABC Kit (Vector Laboratories, California, US) following these incubation protocol: serum 30 min RT, anti-p62 Ick ligand antibody (Cat. 610832, BD Transduction Laboratories, California, US) 1 h RT, 2° biotinylated antibody 30 min RT, ABC reagent 30 min RT, Vector DAB reagent 10 min, HCM (Harry’s haematoxylin 2 min, Scott’s tap water until blue colour, dehydration and clear through 70%/95%/2x100% ethanol/2× xylene, mount in DPX).

In staining for phospho-TDP-43, deparaffinisation, hydration and antigen retrieval were done in a pressure cooker (Antigen Access Unit, A. Menarini, Berkshire, UK) at pH 6 using the Access Citrate solution. Then, the slides were stained using the A. Menarini Intellipath Kit through the following incubation steps: Endogenous peroxidase block 5 min room temperature (RT), casein background blocker 10 min RT, anti-phospho-TDP-43 antibody (Cat. TIP-PTD-M01, Cosmo Bio Co, Tokyo, Japan) 1 h RT, universal probe 15 min RT, HRP-polymer 15 min RT, DAB chromogen 5 min RT, HCM.

### Genome wide association study

ALS susceptibility genes were identified by a large genome wide association study (GWAS) which used the NeuroX chip [32] to genotype 3539 ALS cases and 5191 normal controls; the NeuroX chip includes genotyping of standard Illumina exome content of approximately 240,000 variants, and additionally, more than 24,000 custom content variants to improve coverage in genes associated with neurological diseases. Genes significantly associated with ALS were unchanged when the analysis was performed with the custom NeuroX chip content removed to avoid potential bias. GWA on the NeuroX collaboration was analysed using PLINK [37]. 267607 SNPs were analysed in 10081 founders (0 non-founders identified). No SNPs failed frequency and genotyping pruning. Association with ALS was determined by Chi^2^-test; threshold for significance was set at an unadjusted *p*-value of 5E-08 [4].

Alzheimer’s GWA genes were identified using GWAS Central (http://www.gwascentral.org), which is a compilation of summary level findings from genetic association studies. 57 studies were identified containing the keyword ‘Alzheimer’s’. Variants associated with Alzeimer’s disease at a *p*-value <5E-08 and their associated genes were identified.

### Gene expression data analysis

Microarray data were normalised using the Puma package which quantifies technical variability to improve the estimation of gene expression [27]. The significance of association of transcript expression levels with continuous variables such as pathology counts and disease duration was determined by Spearman rank correlation. Differential expression between two groups was determined by Mann–Whitney U-test. In the identification of significant enrichment of gene list ‘x’ in gene list ‘y’ we utilised Fisher’s exact test to calculate the probability that the observed overlap occurred by chance.

Conversion between various gene/transcript identifiers was performed using Affymetrix Human Genome U133 Plus 2.0 Array annotation data and BioMart [13].

Network detection was performed using weighted gene coexpression analysis (WGCNA) [25]. Global interaction partners of network genes were identified using co-expression and proteomics data from the GeneMANIA prediction server [52].

For the purpose of all analyses in lymphoblastoid cells and in CSF, patients with disease duration <2 years were defined as rapidly progressive and patients with disease duration >4 years were defined as slowly progressive.

### Measurement of soluble TREM2 in CSF

CSF concentrations of sTREM2 were measured using a standard sandwich ELISA consisting of a biotinylated polyclonal goat anti-human TREM2 capture antibody (R & D Systems BAF1828); a monoclonal mouse anti-human TREM2 detection antibody (R & D Systems MAB1828); and a SULFO-TAG–labeled anti-mouse secondary antibody (Meso Scale Discovery R32AC-1). Recombinant human TREM2 protein (huTrem2-hIgG1aglyFc) was produced at Biogen in Chinese hamster ovary (CHO) cells and purified by size-exclusion chromatography to remove aggregates since aggregated proteins can lead to higher binding. Streptavidin-coated 96-well plates (Meso Scale Discovery L15SB) were blocked overnight at 4 °C in blocking buffer [0.5% bovine serum albumin (BSA) and 0.05% Tween 20 in PBS (pH 7.4)]. The plates were then incubated with the capture antibody for 1 h at room temperature. Plates were washed three times with washing buffer (0.05% Tween 20 in PBS) and incubated with the CSF samples diluted 1:4 or with a titration of recombinant human TREM2 protein (2000 ng/ml to 0.1 ng/ml) for 2 h at room temperature. Plates were washed three times with washing buffer before incubation with the detection antibody for 1 h at room temperature. After three additional washes, plates were incubated with the secondary antibody for 1 h at room temperature. All incubation steps were performed with gentle shaking. Plates were washed three times with wash buffer, then the electrochemical signal was developed by adding 2× Meso Scale Discovery Read buffer and the light emission measured using the Mesoscale Discovery SECTOR S 600.

All lumbar punctures were clinically indicated. We aimed to compare levels of soluble TREM2 in CSF from sporadic ALS patients to levels in normal controls. Previously it has been noted that levels of soluble TREM2 can be elevated in a number of inflammatory CNS diseases [35]; therefore our criteria for selection of control cases were: normal CSF constituents and no evidence of neuroinflammation. Diagnoses in control cases included headache with normal CSF, medically unexplained symptoms and cerebrovascular disease. Controls and patients (Table 3) were matched for age and sex; mean age of controls was 51 years (range 34–74 years), mean age of ALS patients was 58 years (range 32–83 years). Controls included 11 males and 9 females; ALS patients included 28 males and 18 females. All samples were collected with written informed consent from the donor, and the work was approved by the South Yorkshire Ethics Committee. Based on the time of sampling relative to disease onset and time of death it was possible to identify at what point in the patients disease course CSF sampling occurred. Early disease was defined as the first 25th-centile of all patients assayed and late disease was defined as the last 25-centile of all patients assayed. In order to minimise the effect of outliers statistical tests were performed using ranks instead of actual values. Correlation with clinical variables was determined by Spearman rank correlation, and differences between groups were determined by Mann–Whitney U-test.

**Table 3:** Clinical information relating to CSF samples obtained from sporadic ALS patients and controls

ALS/Control	Gender	Age at onset (years)	Disease duration (years)	Age at sample (years)	Percentage of disease course at CSF sampling	Soluble TREM2 (ng/ml)
ALS	M	35.8	4.8	36.9	22.8%	309.6
ALS	M	50.7	17.0	53.4	16.2%	2.6
ALS	M	79.7	1.3	79.8	6.3%	10.3
ALS	M	67.4	11.9	72.1	39.2%	10.8
ALS	M	71.5	0.8	72.3	90.0%	5.7
ALS	F	47.8	14.5	49.3	10.9%	3.6
ALS	F	65.3	1.2	66.3	85.7%	7.9
ALS	M	64.4	1.0	65.3	100.0%	6.9
ALS	M	64.3	0.8	64.8	66.7%	2.3
ALS	F	34.4	0.6	34.7	42.9%	4.7
ALS	M	54.4	6.9	56.1	24.1%	9.0
ALS	M	52.6	5.4	54.0	26.2%	27.4
ALS	M	56.4	12.0	57.8	11.8%	4.2
ALS	F	76.2	1.0	76.9	75.0%	4.9
ALS	F	64.3	0.4	64.5	60.0%	8.1
ALS	M	69.4	0.7	69.7	37.5%	37.7
ALS	M	42.9	10.5	43.9	9.5%	5.3
ALS	M	47.1	1.3	47.7	43.8%	18.4
ALS	F	54.2	5.8	54.7	8.7%	14.5
ALS	M	57.7	1.1	58.0	30.8%	14.8
ALS	F	51.2	5.6	52.3	19.4%	22.8
ALS	F	45.2	8.6	46.0	9.7%	8.6
ALS	F	59.5	1.4	59.8	17.6%	9.9
ALS	M	36.8	4.4	38.7	43.4%	8.5
ALS	M	32.0	8.8	37.9	67.0%	18.2
ALS	F	50.7	3.8	51.7	26.7%	2.9
ALS	M	29.8	4.5	31.5	38.9%	2.8
ALS	F	68.5	2.5	69.9	56.7%	4.0
ALS	M	81.0	1.6	82.5	94.7%	4.3
ALS	M	64.3	4.5	67.6	72.2%	25.1
ALS	M	50.6	2.2	51.4	38.5%	16.5
ALS	M	65.1	3.6	67.7	72.1%	9.7
ALS	F	58.7	1.3	59.6	73.3%	16.0
ALS	M	59.2	5.2	62.1	56.5%	9.4
ALS	F	60.5	1.7	61.6	65.0%	24.8
ALS	M	55.7	1.8	56.9	71.4%	16.2
ALS	M	60.1	4.8	62.8	57.9%	9.0
ALS	M	65.5	2.4	66.5	41.4%	5.1
ALS	F	49.9	3.8	52.5	67.4%	17.1
ALS	F	Unavailable	Unavailable	63.8	Unavailable	8.3
ALS	F	52.9	3.4	54.3	41.5%	5.1
ALS	M	50.8	2.6	51.4	25.8%	7.3
ALS	F	62.1	7.7	68.3	80.4%	34.9
ALS	M	32.9	2.6	33.9	38.7%	4.7
ALS	F	47.3	1.7	47.8	30.0%	4.4
ALS	M	66.6	3.9	69.6	76.6%	7.9
Control	M	NA	NA	828	NA	4.0
Control	M	NA	NA	407	NA	6.5
Control	F	NA	NA	717	NA	10.6
Control	F	NA	NA	671	NA	7.0
Control	F	NA	NA	628	NA	2.9
Control	M	NA	NA	573	NA	3.4
Control	F	NA	NA	542	NA	2.6
Control	F	NA	NA	563	NA	4.8
Control	M	NA	NA	455	NA	5.9
Control	M	NA	NA	586	NA	4.8
Control	M	NA	NA	473	NA	14.2
Control	F	NA	NA	587	NA	3.1
Control	F	NA	NA	594	NA	12.9
Control	M	NA	NA	886	NA	9.4
Control	M	NA	NA	782	NA	14.7
Control	M	NA	NA	553	NA	12.3
Control	M	NA	NA	502	NA	5.1
Control	F	NA	NA	701	NA	8.9
Control	M	NA	NA	673	NA	6.7
Control	F	NA	NA	475	NA	3.4

*M* Male, *F* Female

## Results

We aimed to identify a set of genes that can predict ALS disease progression when measured in tissues that are core to disease, but also in tissues that are accessible clinically. Our systems approach has two phases of investigation: a discovery phase (Fig. 1a–c) and a subsequent biomarker assessment phase (Fig. 1d).

### Identifying correlates of neuropathology

To identify genes expressed in correlation with the number of proteinaceous inclusions within motor neurons (pathology correlates), we performed targeted immunohistochemistry and gene expression profiling in ALS motor neurons (Fig. 1a). Cervical spinal cord anterior horn was examined from 11 ALS patients including seven *C9ORF72*-ALS patients and four patients with sporadic ALS (Table 1, patients 1–11). Total RNA was extracted from isolated motor neurons and expression of 54,675 annotated transcripts was measured by microarray analysis. In adjacent tissue from the same patients we counted the number of motor neurons per unit area containing a p62-positive cytoplasmic inclusion (Additional file 1: Figure S1). Spearman rank correlation was calculated between the expression of each transcript and the pathology counts. Eighty-three transcripts, corresponding to 59 genes, correlated with the quantity of pathology (*p* < 0.01) (Additional file 2: Table S1).

Motor neuron inclusions in ALS are expected to stain for TDP-43 and p62 [33]. We confirmed coincidence of p62- and TDP-43-positive staining in cervical cord from the same cases. Presence of p62 and TDP-43 was significantly correlated, despite measurement in non-overlapping tissue sections (Spearman rank correlation, *p* < 0.05, Additional file 1: Figure S2).

### Derivation of gene modules associated with neuropathology

To explore their functional context, each of the 83 pathology-correlated transcripts were used as a seed to identify transcripts with similar expression (top 1% of transcripts by Pearson’s correlation, Fig. 1b). Transcripts were combined into a single network, which was divided into modules of highly correlated genes by weighted gene co-expression analysis (WGCNA) [25, 55]. WGCNA is an established method for module detection within gene co-expression networks and has been previously applied in biomarker development [48]. WGCNA analysis revealed 82 network modules of between 35 and 515 transcripts (Additional file 1: Figure S3, Additional file 2: Table S1). Forty-five network modules contained one or more of the 83 pathology-correlate transcripts. The remaining modules were discarded.

### Systematic prioritisation of gene modules based on enrichment with ALS biology

To identify which of the 45 gene modules have potential as biomarkers we needed an independent and systematic test of their relevance to ALS biology. For this purpose three assessment gene sets were curated to represent rate of progression and upstream genetic association with ALS (Fig. 1c). A motor neuron gene set was generated from laser captured motor neurons (Table 1, patients 1–14); motor neurons were obtained from a set of patients which overlapped but was distinct from the data used to derive the 83 pathology-correlated transcripts. It contained 1705 transcripts significantly correlated with disease duration (*p* < 0.05) (Additional file 2: Table S2). A lymphoblastoid cell gene set was generated from peripherally accessible blood-derived lymphoblastoid cells (Table 2 patients 1–26). It contained 4070 transcripts differentially expressed between patients with rapidly and slowly progressive disease (*p* < 0.05). (Additional file 2: Table S3).

The third assessment set was a genome wide association gene set which consisted of 62 genes containing variants (unadjusted asymptotic association test, *p* < 5E-08) associated with ALS in a genome wide association study (GWAS) of 3539 ALS cases and 5191 normal controls (Additional file 2: Table S4). Genetic variants associated with ALS are by definition upstream. Modules enriched with genetic determinants are more likely to be predictive as genetic determinants are by definition upstream of a disease that occurs in adulthood.

**Fig. 2:**
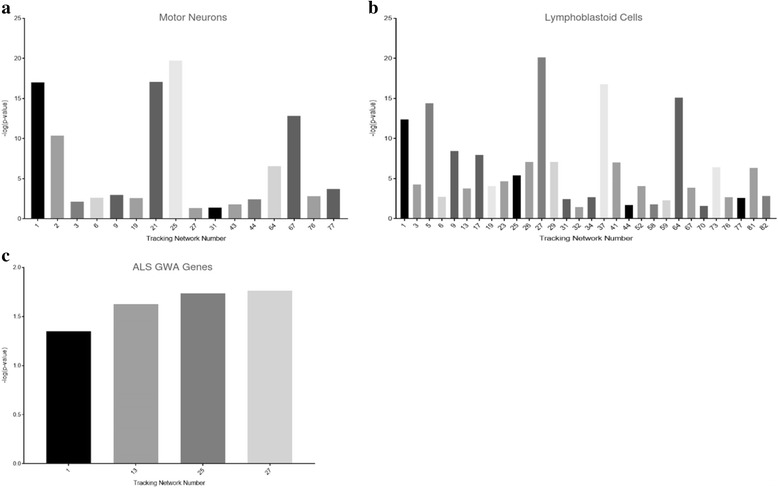
Prioritisation and functional enrichment of genes modules. Gene co-expression modules associated with ALS neuropathology were identified using WGCNA; modules are numbered 1–82. Modules were tested for enrichment with three assessment gene sets curated to represent rate of progression in motor neurons (**a**) and lymphoblastoid cells (**b**), and upstream genetic association (**c**) with ALS. -log (*p*-value) refers to the *p*-value for enrichment of the corresponding module number with the relevant assessment set as calculated by Fisher’s exact test. Only modules significantly enriched with each assessment set (*p*-value <0.05) are plotted with respective *p*-values

Three of the 45 modules were enriched with all three assessment gene sets (Fig. 2, modules 1, 25 and 27). Module 25 was the most highly enriched with the motor neuron gene set (*p* = 1.92E-20) and module 27 was the most highly enriched with the lymphoblastoid gene set (*p* = 7.82E-21). Both module 25 and 27 were significantly enriched with ALS GWA genes (*p* value < 0.05). Module 25 and 27 were selected for further characterisation.

To determine whether modules 25 and 27 captured aspects of disease pathogenesis or simply show motor neuron-specific gene expression, we constructed a negative control from an artificial module consisting of genes which are expressed specifically in non-diseased motor neurons [12] (Additional file 2: Table S1). The negative control module was not enriched with the assessment gene set derived from motor neurons or with ALS GWA genes; there was limited enrichment with the lymphoblastoid gene set (*p* = 0.001). Modules 25 and 27 showed significantly better enrichment with ALS biology gene sets than the negative control module.

### Functional characterisation of modules

As modules 25 and 27 showed significant enrichment with ALS biology gene sets we sought to determine the function of genes in these modules (Fig. 1c). Module 25 enriched for the Gene Ontology (GO) biological process categories ‘synaptic transmission, cholinergic’ and ‘response to oxygen stimulus’ (g: Profiler, corrected *p*-value = 0.04) [40]. Module 27 enriched for the GO category ‘immune system process’ (corrected *p*-value = 7.32E-07). Module 27 will henceforth be referred to as the immune module.

Enrichment within module 27 of immune-associated genes suggests that glial cells proximal to diseased motor neurons may have been laser captured alongside extracted motor neurons. To explore which glial cells might have been isolated we examined cell-specific expression of module 27 genes using a reference database of transcriptome data from isolated human brain cell lines (http://web.stanford.edu/group/barres_lab/brainseqMariko/brainseq2.html). Genes within the immune module were classified as expressed in one or more of microglia/macrophage cells, astrocytes and oligodendrocytes (Additional file 2: Table S5). 61% of the genes in the immune module are known to be expressed in human microglia/macrophage cells as compared to 30% in mature astrocytes and 17% in oligodendrocytes. The majority of immune module genes are expressed in microglia/macrophage cells rather than alternative glial subtypes.

### Development of immune module (module 27) into a peripheral tissue biomarker

A clinically translatable biomarker needs to be measurable in accessible tissue. Markers of inflammation associated with neurodegeneration have been observed in blood [44] and CSF [31]. Module 27 (the immune module) was highly enriched with the assessment set containing genes associated with disease progression in lymphoblastoid cells (*p* = 7.82E-21). We chose to focus on the immune module for biomarker development.

**Fig. 3:**
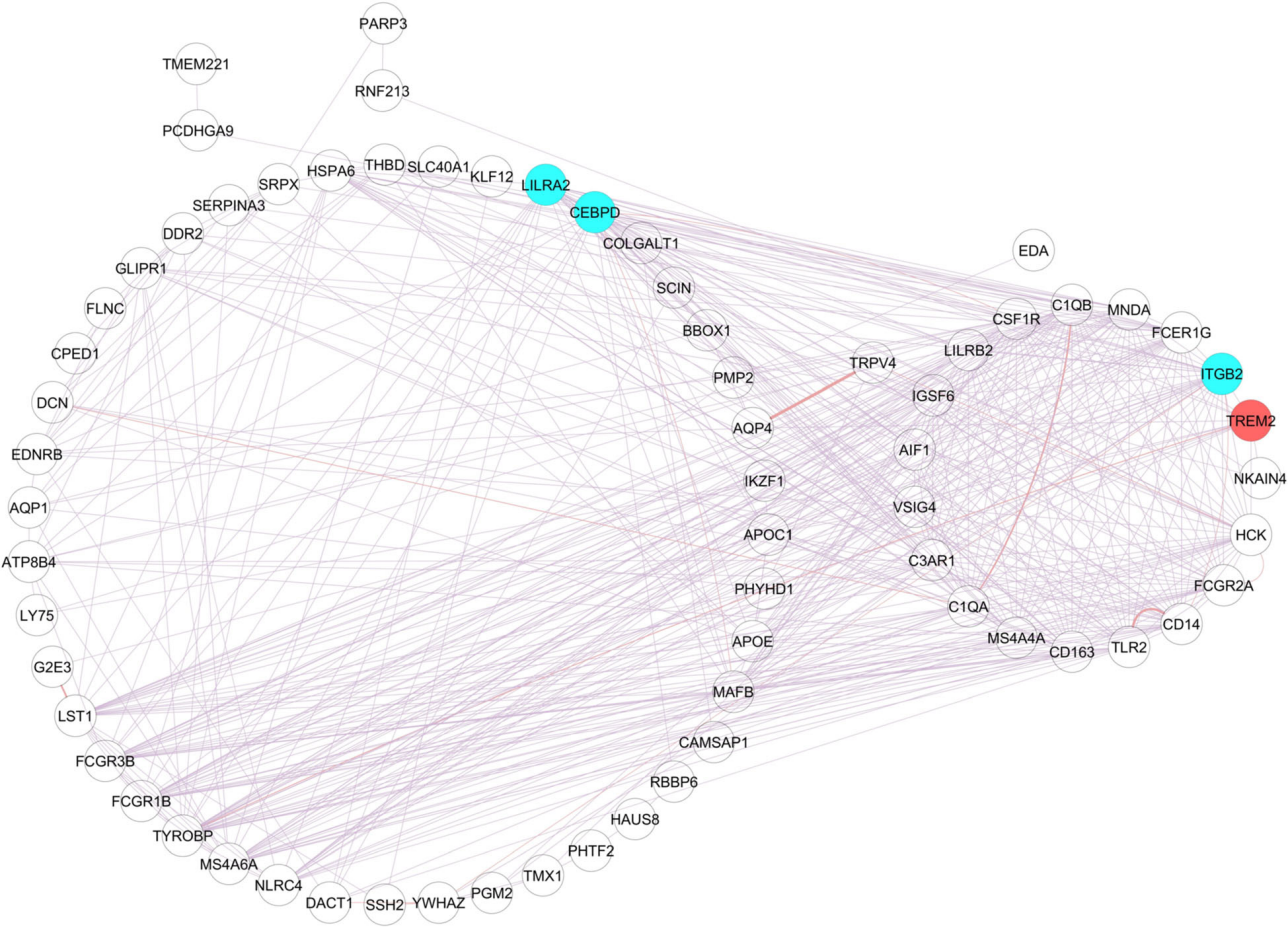
Construction of the immune network independent of cell type by addition of globally co-expressed genes and protein-protein interacting partners. The immune network module (module 27) contained 65 genes which was expanded to 77 genes by addition of globally co-expressed genes and protein-protein interacting partners. Each gene is represented by a node and is labelled with its HUGO identifier. Genes originating from module 27 are arranged on the *left-side* of the diagram; genes identified as globally co-expressed or protein-protein interacting partners are arranged on the *right-side* of the diagram. Relationships between genes are represented as edges between nodes, either global co-expression (*purple*) or protein-protein interaction (*pink*). Only genes with edges reaching statistical significance are shown. *CEBPD*, *LILRA2* and *ITGB2* (*blue nodes*), represent a proposed blood-based biomarker; TREM2 (*red node*) protein m﻿easured in CSF correlates with disease duration in selected patients

To reduce neuron-specific signal and improve likelihood of detecting genes expressed in the immune module in peripheral tissues, we added tissue-independent globally co-expressed genes and protein-protein interacting partners [11] using a database of broadly co-expressed genes with functional association data (GeneMANIA) [52] (Fig. 1d). The immune module was expanded from 65 to 77 genes (Fig. 3). We tested the expanded module against the assessment gene sets representing rate of ALS disease progression and showed an improvement in biomarker performance. The module showed improved enrichment with gene sets related to rate of disease progression in motor neurons gene (*p* = 6.14E-03 from 4.56E-02), and in lymphoblastoid cells (*p* = 1.94E-32 from 7.82E-21).

### Assessment of immune module as a potential biomarker in blood

To provide evidence in support of the immune module as a potential biomarker, we first explored its predictive capabilities in lymphoblastoid cells derived from the blood of *C9ORF72*- and sporadic ALS patients with rapid and slowly progressive disease (Fig. 1d, Table 2). *C9ORF72*-ALS patient samples were used at a previous stage to prioritise the immune module but the sporadic ALS patients comprise an entirely independent dataset. By testing for biomarker performance of the immune module in both datasets separately we aimed to reduce the likelihood of a false positive result.

First we evaluated whether gene expression in the immune module could predict ALS severity as indicated by the time between onset of symptoms and death. Age of onset and sex have been independently linked to prognosis in ALS [38]. Clinical interventions such as artificial respiratory support have also been shown to affect survival but this data was not available. We fitted a Cox proportional hazards model including age of symptom onset, sex and disease duration (to nearest half-year, Additional file 1: Figure S4) together with the top 15 principal components of gene expression in the immune module. In both *C9ORF72* and sporadic ALS, the model was significantly predictive of disease severity (Chi^2^; *C9ORF72*-ALS *p* = 0.01; sporadic ALS *p* = 0.004). To further test the significance of this result we performed an identical analysis using the negative control module representing genes specifically expressed in non-diseased motor neurons. The top 15 principal components of gene expression in the control module were not significantly predictive in either dataset (Chi^2^, *p* > 0.1).

Next, to determine if the module could be useful to support personalised treatment based on classification, we asked whether gene expression in the immune module could effectively classify patients with rapid versus slowly progressing disease. Binomial logistic regression on expression of individual genes within the immune module identified those genes which differentiated lymphoblastoid cells from patients with rapid and slowly progressive disease compared to the null model. Fifteen of the immune module genes differentiated rapid and slowly progressive *C9ORF72*-ALS cases; and in sporadic ALS, 20 genes differentiated rapid and slowly progressive cases (Additional file 2: Table S6). *LILRA2*, *ITGB2* and *CEBPD* (Fig. 3) were predictive in both *C9ORF72*-ALS and sporadic ALS. Fitting binomial logistic regression with leave-one-out cross validation confirmed that a model combining expression of *LILRA2*, *ITGB2* and *CEBPD* was able to correctly classify patients by disease severity more often ﻿than would be expected by chance (85% of *C9ORF72* and 60% of sporadic ALS classified correctly, Additional file 1: Figure S4). Interestingly *LILRA2*, *ITGB2* and *CEBPD* are expressed by microglia/macrophage cells (Additional file 2: Table S5).

### Assessment of immune module as a potential biomarker in CSF

CSF is frequently used to observe CNS-inflammation [31]. We wished to determine if members of the immune module may have potential as a biomarker in CSF. CSF is relatively acellular and therefore suited to a protein-level rather than gene expression quantification. It was not technically feasible to assess all members of the immune module. TREM2, a member of the immune module (Fig. 3), had an available assay and known association with neurodegeneration [20, 34, 36, 47]. We chose to evaluate soluble TREM2 in CSF as a potential biomarker for ALS (Fig. 1d). Concentrations of soluble TREM2, which is cleaved from the surface of microglia [34], have been measured by ELISA in CSF [24, 34]. Genes thought to determine levels of soluble TREM2 in CSF identified by genome-wide complex trait analysis [36] (Additional file 2: Table S7), are enriched in the immune module (Fisher’s exact test, *p* = 0.04).

**Fig. 4:**
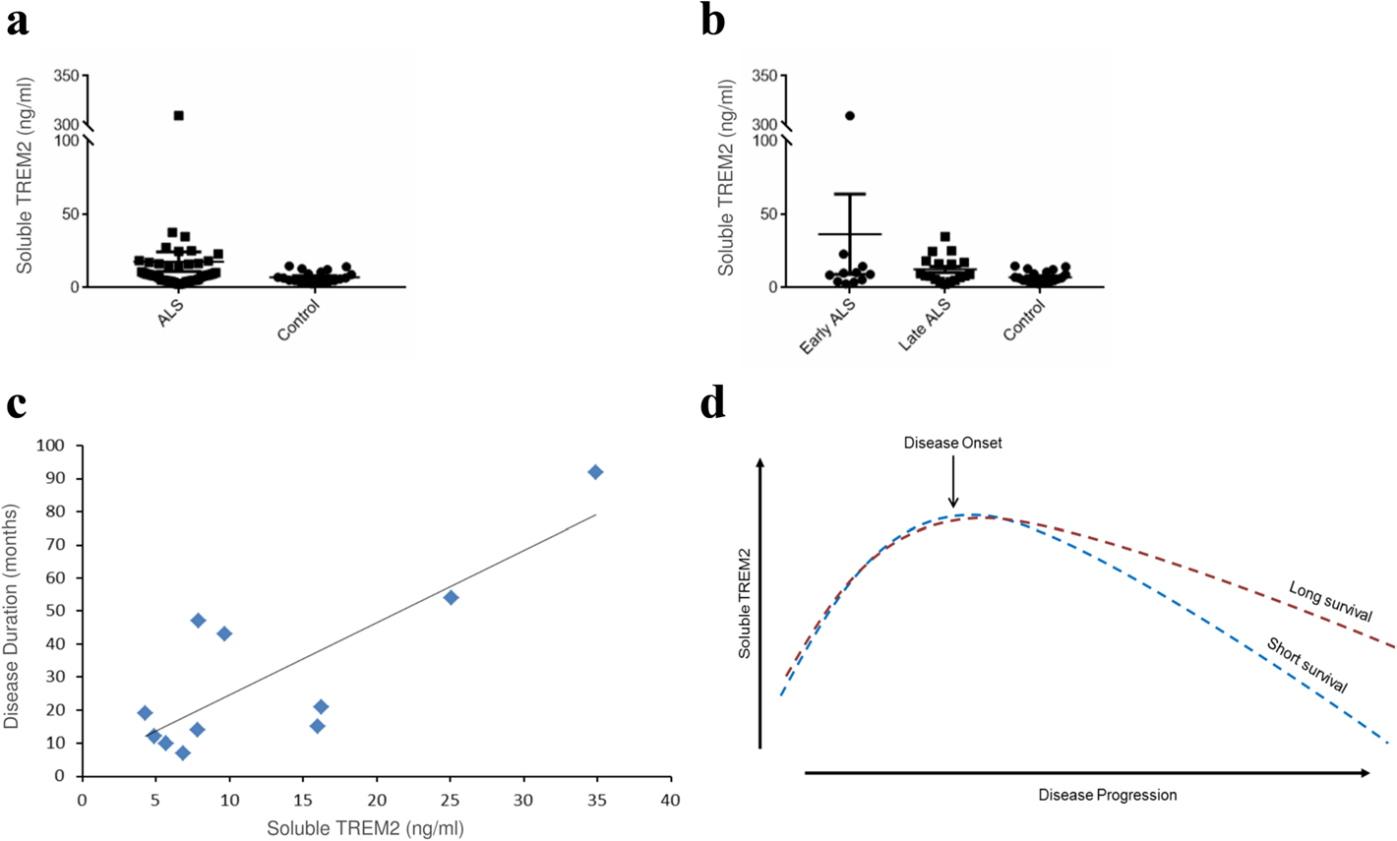
Measurement of soluble TREM2 in CSF from ALS patients and controls. Soluble TREM2 levels were measured by ELISA in CSF from ALS patients (*n* = 46) and controls (*n* = 20) who were age and sex matched. Levels of soluble TREM2 are significantly higher in ALS patients compared to controls (Mann–Whitney, *p* < 0.05) (**a**). Stage of ALS at the time of sample was determined by the time from onset to sample compared to time from onset to death (censored). Levels of soluble TREM2 are highest in early ALS (CSF sampled in <25th centile of disease course), intermediately raised in late (>75th centile of disease course) and lowest in controls (**b**). *Error bars* show standard error. Levels of soluble TREM2 are positively correlated with disease duration in late stage ALS (**c**). We suggest a model whereby CSF soluble TREM2 is elevated in early disease in all ALS patients but then gradually reduces. In certain patients levels remain relatively high reflecting a prolonged neuroprotective microglial activation which leads to slower disease progression (**d**)

Levels of soluble TREM2 were measured in CSF from sporadic ALS patients with varying disease severity (*n* = 46) and controls with normal CSF constituents (*n* = 20) (Table 3). The effectiveness of TREM2 as a biomarker was investigated in two ways; first, we examined whether levels of soluble TREM2 are altered in ALS in comparison to healthy controls, and second, we tested whether soluble TREM2 can classify rapid and slowly progressive ALS. Levels of soluble TREM2 were significantly higher in CSF from ALS patients compared to controls (mean of 18 ng/ml compared to mean of 7 ng/ml, Mann–Whitney *p* = 0.04, Fig. 4a). Levels of measured soluble TREM2 in controls are comparable to other studies [36, 47].

TREM2 has been implicated in stimulation of microglia to clear Alzheimer’s-associated protein aggregates [24]. We tested for enrichment of Alzheimer’s disease GWA genes (Additional file 2: Table S8) within the immune module and found that it is highly enriched (Fisher’s exact test, *p* = 1.83E-07). From this we postulate that the immune module captures a molecular response to neuropathology not just in ALS, but in neurodegeneration more broadly.

In Alzheimer’s disease levels of soluble TREM2 are higher in early phase disease [46, 47]. The same is true in ALS: mean soluble TREM2 levels are three-times higher in early disease compared to late stage disease (mean soluble TREM2 in early disease = 36 ng/ml, mean soluble TREM2 in late disease = 13 ng/ml, Fig. 4b). Strikingly, in late stage disease levels of soluble TREM2 show a significant positive correlation with disease duration (Spearman rank correlation, *p* = 0.01, Fig. 4c). In early disease there is not a significant correlation. Early elevation of TREM2 expression may reflect an initial immune response to deposition of pathological aggregates which declines over time; higher levels of TREM2 in late disease may reflect a sustained neuroprotective microglial response (Fig. 4d).

## Discussion

Our analysis consisted of a data-driven systematic discovery phase leading to discovery of gene modules which were further evaluated in a biomarker assessment phase. In the discovery phase (Fig. 1a–c), transcriptome-wide gene expression changes in proportion to the development of cytoplasmic proteinaceous inclusions in ALS motor neurons allowed us to discover molecular determinants of disease severity. Gene expression and pathology counts were carried out in the same cell population to avoid confounding by variation between populations. The extent of pathology varies between neuronal populations even within individual patients [3]. Transcripts found to be expressed in proportion to the development of neuropathology were utilised to produce 45 modules of co-expressed genes. In a systematic filtering process these modules were then prioritised by demonstration of enrichment with independent measures of ALS biology. We discovered two gene modules strikingly enriched with gene sets associated with rate of ALS progression in both motor neurons and lymphoblastoid cells, and also with ALS GWA genes.

In the biomarker assessment phase (Fig. 1d) we selected one of the top scoring modules which showed the highest enrichment with rate of progression genes in lymphoblastoid cells, and was enriched with genes associated with immune function. The majority of genes within this module are expressed in microglia as opposed to other glial subtypes. Microglia are crucial for clearance of protein aggregates [16, 51] which is biologically consistent with our focus on motor neuron pathology. Many genes within the immune module have not been previously implicated in ALS, however others have highlighted the role of neuroinflammation and microglial activation in disease progression [10, 44, 45] making this module a good candidate for further investigation. Given that CNS immune function can be observed peripherally [18, 31], we tested the potential of this module to be a prognostic biomarker in peripherally accessible tissue.

In tissue derived from patient blood, we demonstrated that expression of the immune module as a whole was significantly associated with ALS disease duration. Moreover, a three-gene panel comprising *LILRA2*, *ITGB2* and *CEBPD* was found to correctly classify individuals as suffering from rapid or slowly progressive disease, independent of both genetic background and clinical intervention such as respiratory support. Measurement in a relatively small number of patients relying on microarray technology is a limitation of these data but a larger biomarker validation study is beyond the scope of this study.

CSF is also peripherally accessible. *TREM2* is a member of the immune module which has been previously linked to both ALS pathogenesis [5] and microglial activation [6]. We investigated the potential for soluble TREM2 in CSF to predict disease course in ALS patients with mixed genetic background. Soluble TREM2 cleaved from the surface of microglia has been proposed as a biomarker in other neurological diseases including Alzheimer’s disease and multiple sclerosis [20, 34, 36, 47]. We show that soluble TREM2 levels are significantly elevated in ALS compared to controls. Elevation is most marked in early disease, as has been observed in Alzheimer’s disease [46, 47]. Importantly, in patients where CSF was acquired in late stage disease, higher concentrations of soluble TREM2 are strongly associated with slower disease progression. Marked early elevation of TREM2 expression may reflect an initial immune response to deposition of pathological aggregates which declines over time. It is hypothesised that patients with higher levels of TREM2 in late disease have mounted a sustained neuroprotective microglial response (Fig. 4d).

Loss-of-function (LOF) mutations in *TREM2*, which have been linked to risk of Alzheimer’s disease [7, 23], and ALS [5], reduce phagocytosis of aggregated protein by microglia [24]. Reduced phagocytosis may be toxic to stressed neurons and indeed TREM2 activity has been positively associated with a neuroprotective microglial phenotype [39]. Modulating microglial activity through TREM2 has been proposed as a therapeutic target in Alzheimer’s disease [53]. Our data suggests that this therapeutic strategy may also be applicable in ALS. In addition to TREM2, it is probable that our immune module contains other determinants of neuropathology relevant to neurodegeneration more broadly: consistent with this the immune module is enriched with GWA genes for both ALS and Alzheimer’s disease.

## Conclusions

The role of microglia in neurodegeneration is controversial. There is evidence for microglia mediated neurotoxicity and neuroprotection. For example, [11C](R)-PK11195 positron emission tomography assay of microglial activation in motor cortex is positively correlated with burden of upper motor neuron degeneration [49] but compromised microglial function through LOF mutations in TREM2 increases the risk of ALS [5]. To explain this controversy microglia are thought to be capable of multiple phenotypes which are variably neuroprotective and neurotoxic [1]. Our immune module is derived by association with motor neuron pathology and predicts ALS prognosis. The prevalence of microglial-expressed genes within this module supports the possibility that there is a direct link between microglial function and motor neuron death. The positive correlation we have identified between soluble TREM2 concentration in CSF and a more benign ALS phenotype supports the possibility of neuroprotection mediated by microglial phagocytosis.

We have performed a scalable systematic, objective discovery of potential predictive biomarkers and a potential therapeutic target. Pathology-correlated gene expression in motor neurons has, for the first time in a data-driven manner, identified microglial function as an important determinant of ALS pathogenesis across a broad spectrum of genetically heterogeneous patients who all display TDP-43/p62 proteinopathy. Microglia are implicated in neurodegenerative disease [10, 45] and are thought to be responsible for clearance of protein aggregates, clearly linking them to development of neuropathology [16, 51]. We propose that phagocytosis of protein aggregates by microglia is likely to be therapeutic and enhanced by TREM2 signalling, making phagocytosis of protein aggregates by microglia an important focus for future translational research in ALS and other neurodegenerative diseases.

## Additional files

Additional file 1: Figures S1–S4. Contain plots of pathology counts in ALS-motor neurons and details of the WGCNA analysis used to derive network modules. (PDF 723 kb)
Additional file 2: Tables S1–S8. Contain gene lists used in the analysis. (XLSX 317 kb)

## Abbreviations

ALS: Amyotrophic lateral sclerosis
CHO: Chinese hamster ovary cells
CNS: Central nervous system
CSF: Cerebrospinal fluid
GO: Gene Ontology
GWAS: Genome wide association study
RT: Room temperature
WT: Whole transcript
