# Supplementary material for: A data-driven approach links microglia to pathology and prognosis in amyotrophic lateral sclerosis
Source: Acta Neuropathol Commun. 2017 Mar 16;5:23. doi: 10.1186/s40478-017-0424-x (PMC5353945; doi:10.1186/s40478-017-0424-x)
Supplement: Additional file 1: Figures S1–S4. — Contain plots of pathology counts in ALS-motor neurons and details of the WGCNA analysis used to derive network modules. (PDF 723 kb) [file 40478_2017_424_MOESM1_ESM.pdf]

Supplementary Figure 1: Counts of Motor Neurons Per Unit Area of Cervical Spine Anterior Horn Containing a P62-Positive Cytoplasmic Inclusion

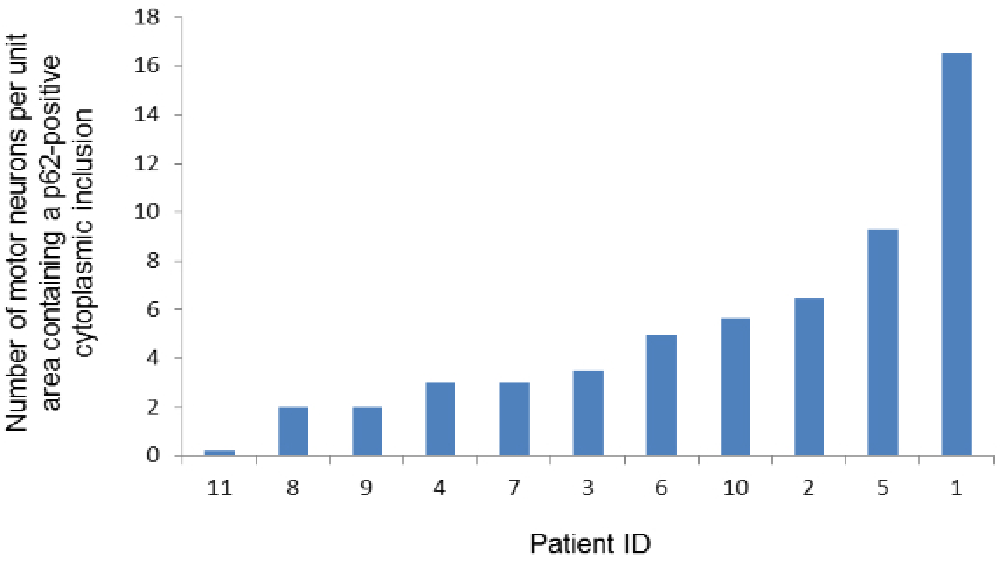

We counted the number of motor neurons per unit area containing a p62-positive cytoplasmic inclusion, in cervical spinal cord anterior horn from 11 ALS patients including seven *C9ORF72*-ALS patients and four patients with sporadic ALS. Counts are plotted for each of cases 1-11 (Table 1).

Supplementary Figure 2: p62- and TDP-43-positive inclusion counts in motor neurons from the same cases are correlated

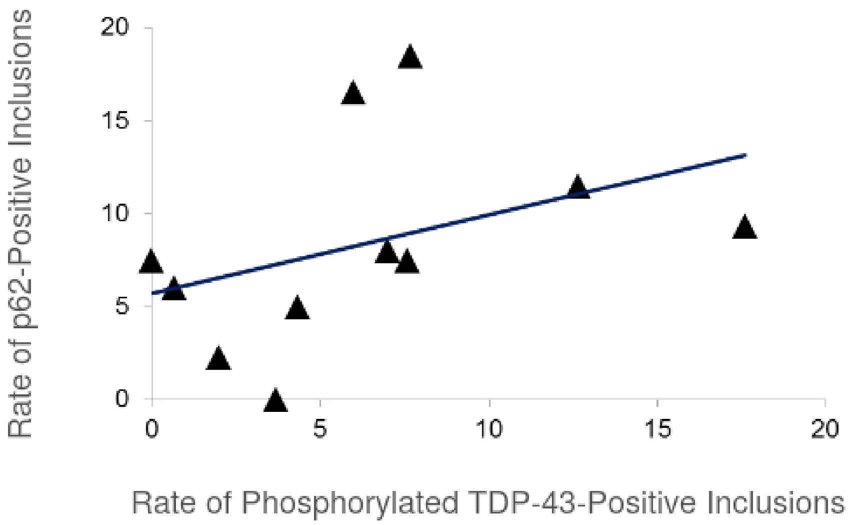

Within independent tissue sections from cervical spine of ALS cases, counts of p62-positive and phosphorylated-TDP-43-positive inclusions are significantly correlated (Spearman rank correlation, $p<0.05$) .

Supplementary Figure 3: Derivation of gene-network modules associated with ALS neuropathology

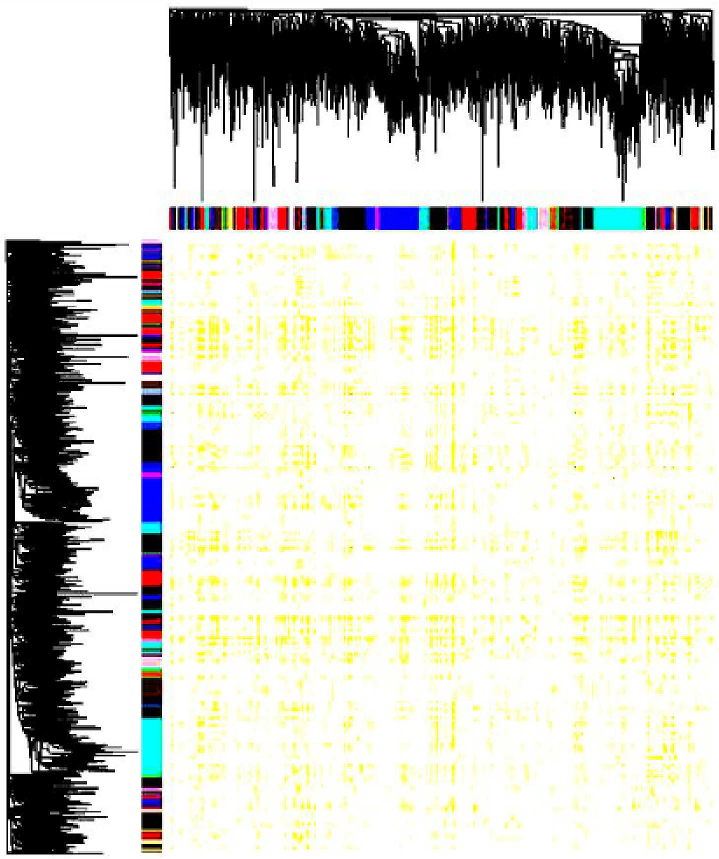

WGCNA analysis identified 82 network modules from genes correlated with counts of proteinaceous inclusions in diseased motor neurons. Clustering tree and heat map illustrate separation of the gene modules, a lower branch height or darker colour denotes a greater Pearson correlation coefficient between pairs of genes.

Supplementary Figure 4: Survival analysis in lymphoblastoid cells from C9ORF72- (n=26) and sporadic ALS (n=20)

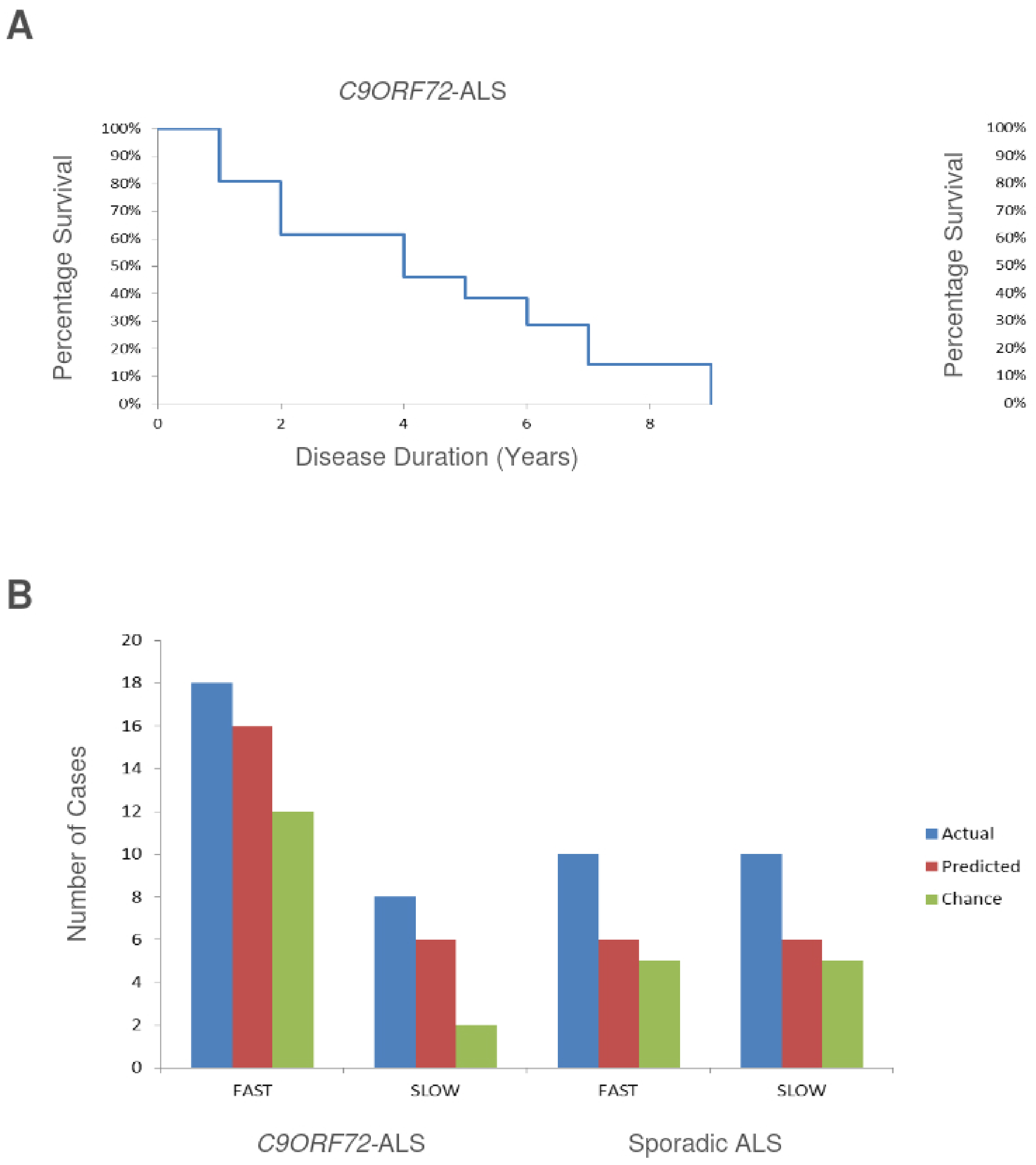

Survival curves for patients from which lymphoblastoid cells were sampled (A). Patient samples were chosen to represent the extremes of survival in ALS. Logistic regression on disease duration identified genes from the immune module capable of classifying patients by rate of disease progression. Fitting binomial logistic regression with expression of ITGB2, CEBPD and LILRA2 and performing leave-one-out cross validation reaches significance for classification of individual patients as rapidly progressive (disease duration <2 years, labelled FAST) or slowly progressive (disease duration >4 years, labelled SLOW) independent of genetic background (B). For each sample group columns depict, from left to right, the number of samples in the group, the number correctly classified by the model and the number of correct classifications expected by chance.
